# Altitude‐mediated soil properties, not geography or climatic distance, explain the distribution of a tropical endemic herb

**DOI:** 10.1002/ece3.8572

**Published:** 2022-02-09

**Authors:** Jacob K. Moutouama, Orou G. Gaoue

**Affiliations:** ^1^ 247620 Department of Ecology and Evolutionary Biology University of Tennessee Knoxville Tennessee USA; ^2^ Faculty of Agronomy University of Parakou Parakou Benin; ^3^ 247620 Department of Geography, Environmental Management and Energy Studies University of Johannesburg Johannesburg South Africa

**Keywords:** abiotic drivers, abundance center distribution, Africa, altitudinal effects, center‐periphery hypothesis, functional traits, interspecific competition

## Abstract

Understanding the ecological processes that govern species' range margins is a fundamental question in ecology with practical implications in conservation biology. The center‐periphery hypothesis predicts that organisms have higher abundance at the center of their geographic range. However, most tests of this hypothesis often used raster data, assuming that climatic conditions are consistent across one square km. This assumption is not always justified, particularly for mountainous species for which climatic conditions can vary widely across a small spatial scale. Previous studies rarely evenly sample occurrence data across the species' distribution. In this study, we sampled an endemic perennial herb, *Thunbergia atacorensis* (Acanthanceae), throughout its range in West Africa using 54 plots and collected data on (a)biotic variables, the species density, leaf mass per area, and basal diameter. We built a structural equation model to test the direct and indirect effects of distance from geographic and climatic niche centers, and altitude on *Thunbergia* density as mediated by abiotic and biotic factors, population demographic structure, and individual size. Contrary to the prediction of the center‐periphery hypothesis, we found no significant effect of distance from geographic or climatic niche centers on plant density. This indicates that even the climatic center does not necessarily have optimal ecological conditions. In contrast, plant density varied with altitudinal gradient, but this was mediated by the effect of soil nitrogen and potassium which had positive effect on plant size. Surprisingly, we found no direct or mediating effect of interspecific competition on plant density. Altogether, our results highlight the role of geography, climatic, and ecological mismatch in predicting species distribution. Our study highlights that where altitudinal gradient is strong local‐scale heterogeneity in abiotic factors can play important role in shaping species range limits.

## Graphical abstract

We tested the center‐periphery hypothesis in *Thunbergia atacorensis*, a range‐limited species and find that plant abundance did not vary with distance from geographic or climatic niche center but decreased with altitude indicating that even climatic centers do not necessarily have optimal ecological conditions. Our study highlights the relative role that abiotic factors play in shaping species range limits and the critical role of altitudinal gradient. Our findings further suggest that to understand range limitation, one must look beyond plant abundance and consider alternative responses such as plant demography and population dynamics.

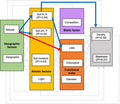

## 1 INTRODUCTION

Understanding the evolutionary and ecological processes that govern species range margins is a central goal in ecology (Sutherland et al., 2013). The center‐periphery hypothesis (CPH) predicts that species are most abundant at the center of their distribution and decline in abundance toward the edge of their distribution (Brown, 1984). One assumption underlying the CPH is that the center of the geographical range coincides with preferred habitats of the species, where ecological conditions are optimal compared to the edge, where conditions are less suitable (Parsons, 1991). This is particularly true when the species' response curve along environmental gradients is unimodal and symmetrical in their tails for all the variables determining the niche (Yañez et al., 2020). Such ecologically optimal conditions will favor higher population density either directly via improved growth, survival, and/or fertility (Greiser et al., 2020) or indirectly via gene flow (Kirkpatrick & Barton, 1997). Recent studies shown that a mismatch between geographic and ecological niches is more likely among species that are poorly dispersed or organisms that are resilient and can persist in poor habitats (Pagel et al., 2020; Schurr et al., 2012).

Two decades ago, Sagarin and Gaines (2002) showed that 61% of studies that empirically or theoretically tested the center‐periphery hypothesis failed to find support for this hypothesis. However, the authors cautioned that only two of the studies included in their analysis sampled the entire species' range, and in most studies, range edges were “severely under‐sampled.” Moreover, most studies used only the geographic distance from range center, which itself does not explain the range of climatic conditions across the species distribution area. With the increase popularity of ecological niche modeling, it is now possible to identify species preferred sites (most suitable areas across the range). Therefore, one could use both the ecological and the geographic marginality gradient to test the center‐periphery hypothesis. Studies that have done this found that species are often more abundant at more suitable sites (Weber et al., 2017) and that species abundance is associated with the ecological, rather than the geographic gradient (Martínez‐Meyer et al., 2013; Yañez‐Arenas et al., 2012).

A recent review by Dallas et al. (2017) used data across taxa to show no change in abundance with distance from their geographic range or climatic niche center. Another synthesis reports that only 51% of studies showed decreased abundance from geographic center to periphery (Pironon et al., 2017). These authors suggested that the lack of support for the CPH is due to the lack of overlap between geographic and climatic ranges. Given such inconsistent patterns in the center‐periphery distribution, a process‐based approach is necessary to examine the variation of not just species abundance but also demography to improve our understanding of the mechanism behind range limitation (Sexton et al., 2009). This includes investigating the center‐periphery variation in functional traits, population demographic structures, and dynamics (Angert, 2009; Pironon et al., 2015; Treurnicht et al., 2020).

Functional traits determine the response of organisms to environmental drivers. These traits are good predictors of species abundance (Li et al., 2021). For example, in grassland communities, plant height and leaf mass per area can explain variation in species abundance (Lisner et al., 2021). In tropical forests, studies have shown that maximum plant height and leaf area were positively correlated with species abundance (Yan et al., 2013). Species traits could therefore determine whether species are most abundant at the center of their distribution. For instance, in North American bird populations, body mass, migratory status, and habitats affected the abundance center‐relationship (Osorio‐Olvera, Yañez‐Arenas, et al., 2020). However, few studies have investigated how functional traits can mediate the center‐periphery distribution of species abundance (but see Treurnicht et al., 2020).

Population demographic structures can vary across species distribution range from center to periphery (Gerst et al., 2011). Skewness measures the asymmetry of the population demographic structure, indicating if the population is dominated by young or mature individuals. Skewness of the trait distribution can be used to understand environmental–trait relationships (Wool, 1980). For example, the distribution of species traits is expected to be symmetrical in suitable conditions and asymmetrical in stressful environmental conditions (Fraser, 1977). Similarly, in central populations where more suitable ecological conditions are expected, one may expect symmetric (skewness = 0) population size distribution. Peripheral populations with less ideal environmental conditions are expected to have more skewed population structures.

Species interactions can shape their range limits (Louthan et al., 2015; Nottebrock et al., 2017; Stanton‐Geddes et al., 2016). Due to species‐specific energy costs and gains in response to environmental conditions, competition can have more impact at the edge than at the center, thereby preventing species expansion beyond range limits (Hall et al., 1992). Further, competition can synergistically interact with abiotic factors to decrease population density at the edge of a species' range (Pulliam, 2000; Vergeer & Kunin, 2013). However, most studies investigating the center‐periphery hypothesis focused on either abiotic or biotic factors, rarely studying the synergistic effects of both drivers. This ultimately limits our mechanistic understanding of the relative roles of abiotic and biotic factors in shaping species ranges. This limitation has implications for both the conservation of rare species and control of invasive species. Even when abiotic factors are included in estimating the climatic niche, the predictor variables used are often rasters collected from WorldClim (see Dallas et al., 2017) and Osorio‐Olvera, Lira‐Noriega, et al. (2020), Osorio‐Olvera, Yañez‐Arenas, et al. (2020). These rasters are derived from monthly temperature and precipitation variables at 1km by 1km or larger scale, with the assumption that abiotic factors are homogenous across the 1km^2^. This assumption is rarely true in mountainous areas where abiotic conditions often vary at a smaller scale (Egli & Poulenard, 2016). Such microscale ecological changes can outweigh the macroclimatic influence on species distribution. It is therefore important to test the center‐periphery variation in species abundance not just considering geographic position or climatic niche variation but also altitudinal gradient particularly for species that occur in mountainous habitats.

In this study, we tested the predictions of the center‐periphery hypothesis by evaluating population density and distance from the center of the geographic range and the climatic niche. We collected biotic and abiotic variables including, altitude, species' density, leaf mass per area, individual size in 54 plots in 12 populations of an endemic perennial herb in West Africa, *Thunbergia atacorensis* (Acanthanceae), monitored throughout its entire range. We predicted that plant density will be shaped by climatic and altitudinal gradients that affect plant functional traits and population structure. We used structural equation modeling (Grace et al., 2010; Shipley, 2000) to examine how variation in soil properties, light availability, and interspecific competition can mediate the effects of altitude, geography, and climatic gradients on plant size, functional traits, population demographic structure, and ultimately plant density from range center to periphery.

## 2 METHODS

### 2.1 Study system

**FIGURE 1:**
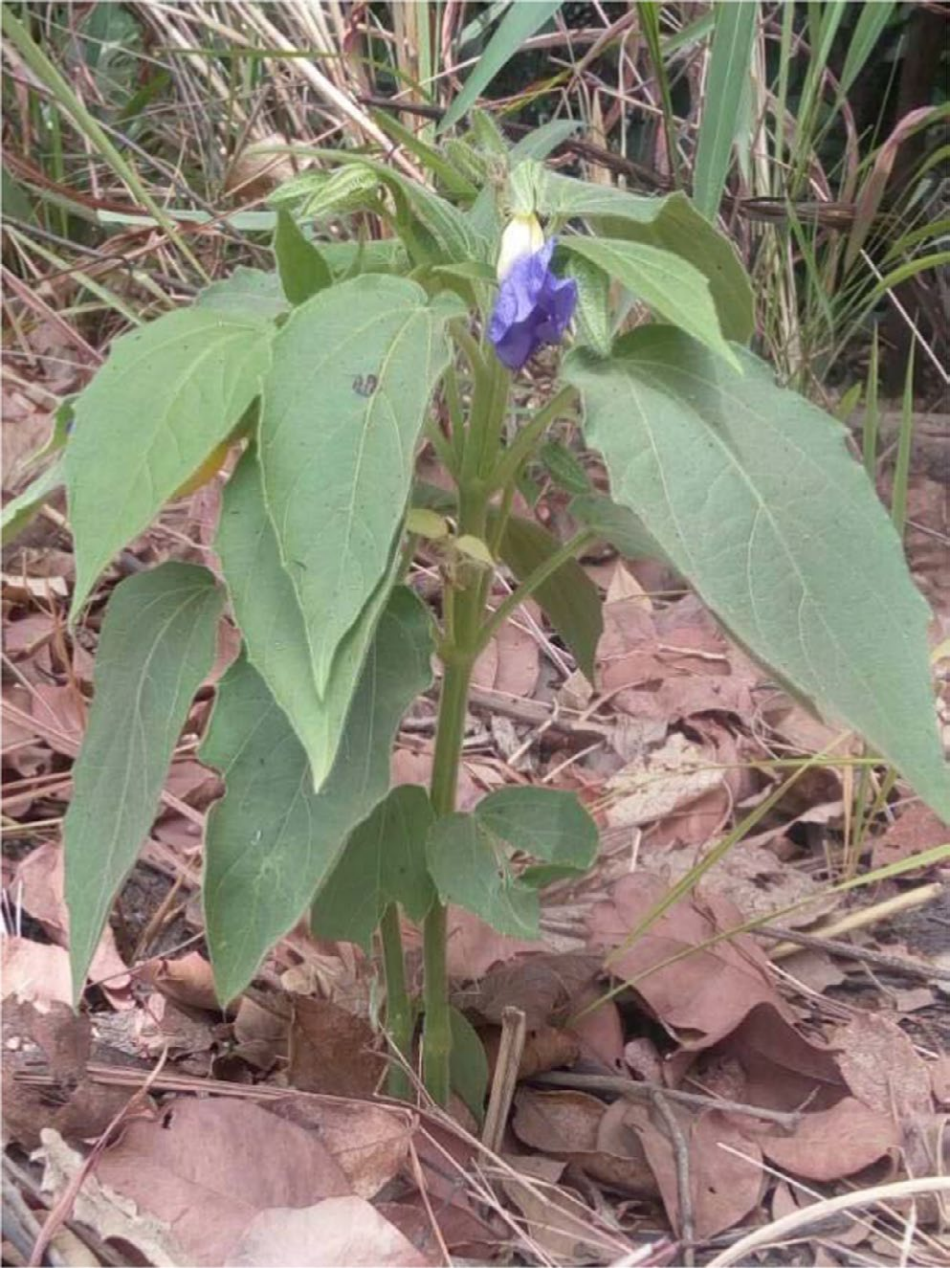
A *Thunbergia atacorensis* adult plant showing a characteristics purple flower in one of our study sites in Benin (West Africa). The species is found in gallery forest along the Atacora mountain chain

We tested the center‐periphery hypothesis using *T*. *atacorensis* (Acanthaceae), a range‐limited perennial herb endemic to West Africa, one of the least studied regions of the tropics. *Thunbergia atacorensis* (Akoègninou Lisowski & Sinsin) is an endemic herb that can reach up to 80 cm in height and is found in gallery forests (Figure 1) along the Atacora mountain chain (Akoègninou et al., 2006) and the Sobakperou mountain in Benin (Fandohan et al., 2015). *Thunbergia atacorensis* reproduces both sexually (Akoègninou et al., 2006) and asexually (Asseh et al., 2017). The species produces flowers and fruits from February to April and September to November (Akoègninou et al., 2006).

**FIGURE 2:**
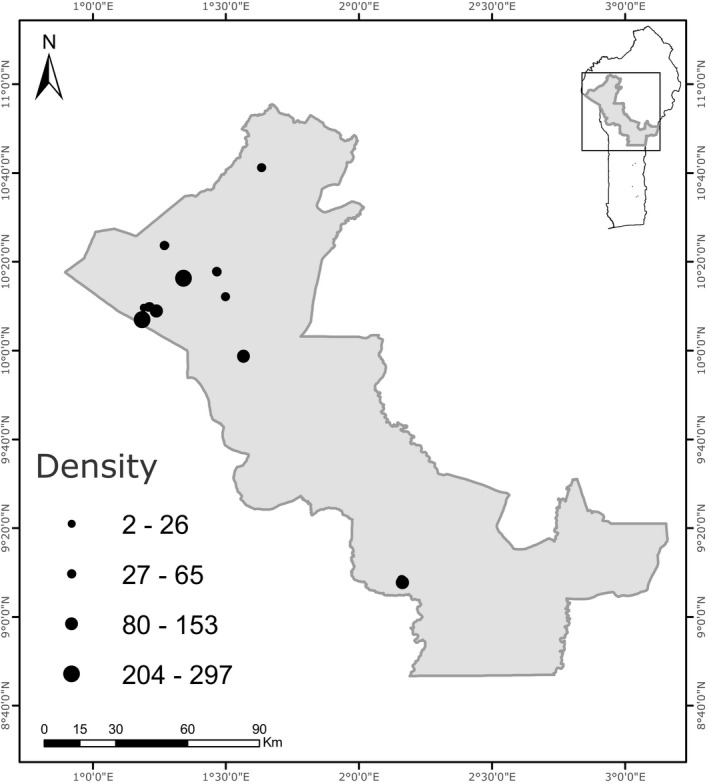
Distribution of 12 *Thunbergia atacorensis* populations in Benin. The gray square in the insert represents the study area in Benin (West Africa). The black dots represent sampled populations. The size of each dot is proportional to population density (individuals/25 m^2^)

We identified and studied all 12 known populations of *T*. *atacorensis* to test the center‐periphery hypothesis (Figure 2). These populations were geographically isolated into two disjoint subgroups, ten populations were located in the Atacora mountains (1°00′–2°00′E and 10°40′–11°28′N) in northwest Benin and two populations in the Sobakperou mountains (2°9′N–9°8′E) in central Benin. Across these regions, the annual rainfall varies from 1200 to 1350 mm and the annual temperature is 28°C (Sinsin & Kampmann, 2010). We established a total of 54 permanent plots in these populations. At each population, we randomly established five 5 × 5 m permanent plots for demographic studies and to estimate plant density. Because three of the populations were small, we installed a minimum of two plots instead of five (see Appendix S1, Table S1).

### 2.2 Plant density, functional traits, competition, and abiotic factors

In each plot, we estimated *T*. *atacorensis* density by counting the number of individual plants per plot. For each plant, we measured the basal stem diameter (using a digital caliper) as a metric of size, leaf chlorophyll content, and estimated leaf mass per area. On each plant, we used a Konica Minolta SPAD‐502Plus Chlorophyll Meter to measure chlorophyll content from three leaves randomly chosen and averaged the values. These three leaves were collected to estimate their area using “LeafByte” (Getman‐Pickering et al., 2019) and later oven‐dried to measure their dry mass. We estimated the leaf mass per area (LMA) as the ratio of leaf dry mass to leaf area. We calculated the skewness of *T*. *atacorensis* basal diameter distribution for each population, using the R package “e1071*”* (Meyer et al., 2019).

To account for abiotic conditions, we measured plot‐level light as the photosynthetic active radiation (PAR) using a Cl‐110 Plant Canopy Imager (CID Bio‐Science Inc., Camas, WA, USA). In each plot, we also measured soil moisture using a Extech MO750 soil moisture meter (Extech, Boston, MA, USA). We then collected 20g of composite soil samples per plot by mixing soil collected at seven inches depth from the center and the four corners of each plot. In total, we collected 54 soil samples, which were analyzed for macronutrients (nitrogen, potassium, phosphorus, sulfur, and magnesium), micronutrients (Iron, Zinc), and pH by the Soil, Water & Plant Testing Laboratory at Colorado State University (CO, USA). To reduce the number of soil variable to consider in our analysis, we performed a principal component analysis (PCA) on soil macronutrients and micronutrients variables using the package “FactoMineR” (Le et al., 2008). The PCA summarized soil variables into five principal components, each representing a unique weighted combination of variables. We used the first two principal components (PC) which explained >50% of observed soil variation (see Appendix S3, Figure S1) as metric of soil fertility. The first PC captures increasing concentrations of nitrogen, potassium, and the second PC captures increasing pH and phosphorus concentrations.

To measure interspecific competition for each plot, we recorded every single plant species found in the plot and measured the maximum height for each species and estimate each species percent cover. We calculated the space resource utilization (SRU), a proxy of species competitive ability for light (Zhang et al., 2015), as $\text{SRU}=\sum_{i}^{n}H_{i}C_{i}A$, where *H_i_* is the average maximum height of species *i*, *C_i_* represents the percent cover of species *i* in a plot, *n* the total number of species per plot and *A* is the plot area.

### 2.3 Estimation of the distance from the geographic center and climatic center

We identified the geographic center of *T*. *atacorensis* by first projecting the geographic coordinates of the twelve populations of *T*. *atacorensis* on a map, using the packages “*sp*” (Pebesma & Bivand, 2020) and “*rgdal*” (Hijmans, 2019) in *R* (R Core Team, 2019). Then, we determined the convex polygon formed by all the 12 populations with the *geosphere* package (Hijmans, 2019). The geographic center was estimated as the centroid of this convex polygon using the function *centroid* in “*geosphere*.” Finally, we estimated the geographic distance of each *T*. *atacorensis* population from the centroid of the convex polygon.

In addition to the geographic center, we identified the climatic niche center and estimated the distance of each population to this center. Unlike the geographic distance, the “climatic distance” measures how far the climatic conditions of a given population are from the optimum climatic conditions of the species. The further the distance, the worst climatic conditions the population is expected to experience. We estimated the climatic distance as a Mahalanobis distance (Osorio‐Olvera, Lira‐Noriega, et al., 2020; Osorio‐Olvera, Yañez‐Arenas, et al., 2020). We preferred the Mahalanobis distance because it takes into account the covariance among variables and therefore corrects the problem of scale and correlation inherent in most other methods used to estimate the climatic distance (Calenge et al., 2008; Etherington, 2019; Farber & Kadmon, 2003). To estimate the Mahalanobis distance, we used the following steps. First, we built a minimum volume ellipsoid model. We combined occurrences data of *T*. *atacorensis* with noncorrelated bioclimatic variables. Specifically, we used 80% of *T*. *atacorensis* occurrence points to calibrate the ellipsoid model, 20% to test the ellipsoid model, and eight noncorrelated bioclimatic variables. The bioclimatic variables were annual mean temperature, mean diurnal range, isothermality, temperature seasonality, maximum temperature of warmest month, annual precipitation, precipitation of driest month, and precipitation of warmest quarter. These bioclimatic variables were derived from spatial interpolation of monthly average temperature (maximum and minimum) and precipitation (total) throughout 1970–2000 for the study region (Fick & Hijmans, 2017) and had a spatial resolution of 30 arc‐second. Second, we estimated the centroid of the minimum volume ellipsoid model using the package “*ntbox*” (Osorio‐Olvera, Lira‐Noriega, et al., 2020). Finally, we used the *mahalanobis* function in the package “*stats”* to calculate the Mahalanobis distance from the centroid of the minimum volume ellipsoid to each plot (Bolar, 2019).

### 2.4 Data analysis

We developed structural equation models (SEM) to test the center‐periphery hypothesis and the mediating role of processes related to soil, competition, and altitude (Lefcheck, 2016). Structural equation modeling is a robust statistical modeling technique that fits network of hypotheses to data (Grace et al., 2010; Shipley, 2000). We developed two meta‐models for our SEM: one using the geographic distance and the other climatic distance (Appendix S4, Figure S1) as the main predictor of *T*. *atacorensis* density. For each meta‐model, we tested the direct and indirect effects of altitude and distances on plant density and population structure skewness as mediated by abiotic factors, including soil fertility, soil moisture, and light exposure. We also tested for the mediating role of biotic factors (interspecific competition) and functional traits like leaf mass‐per‐area, basal diameter, and chlorophyll content. We built structural equation models using the package “piecewiseSEM” (Lefcheck, 2016) that incorporates linear mixed‐effect models with our study populations as random effects (Bates et al., 2015). We used the *d*‐*separation* test to evaluate whether any nonhypothesized, independent relationships were significant and whether including a missing path could improve the model (Shipley, 2013). We reported the conditional variance explained (*R* ^2^) for each response variable included in our final SEM. The conditional *R* ^2^ explains the proportion of variance explained by both fixed and random factors in the mixed effect models (Nakagawa & Schielzeth, 2013). We log‐transformed plant density and standardized all quantitative predictors to a mean of 0 and a variance of 1, to compare the path coefficients. All analyses were performed in R 3.6.2 (R Core Team, 2019) and the data used for this analysis are available online (Moutouama & Gaoue, 2022).

## 3 RESULTS

**FIGURE 3:**
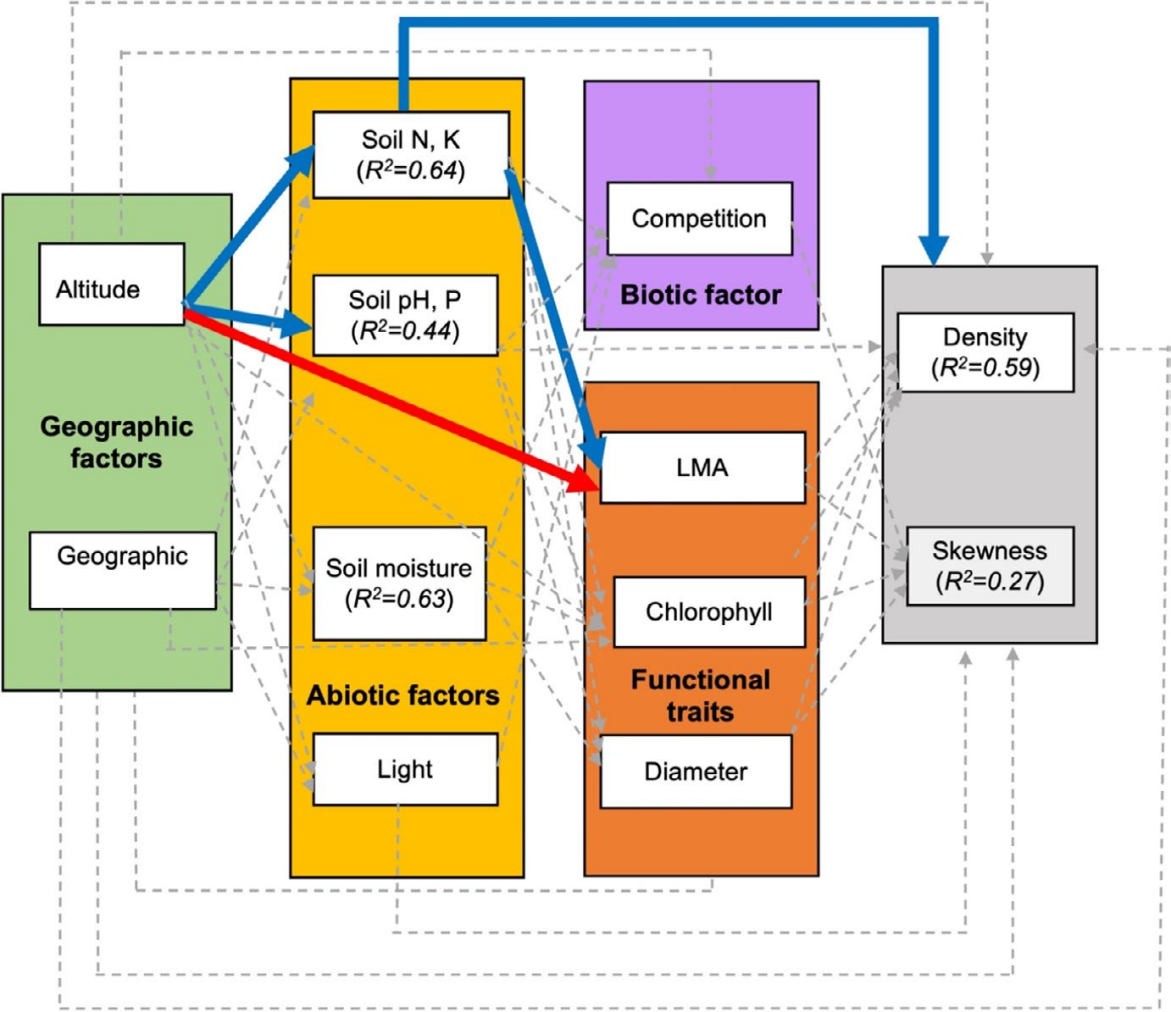
Structural equation model showing how the distance from the geographic center and altitude drives *Thunbergia atacorensis* population density and population structure. Dashed arrow shows nonsignificant correlation, blue arrow means positive correlation, and red arrow shows negative correlations. *R* ^2^ represent the conditional coefficients of determination for each linear mixed effect. Soil N, P represent the first component of the PCA of soil fertility, while Soil pH, P represent the second component

**FIGURE 4:**
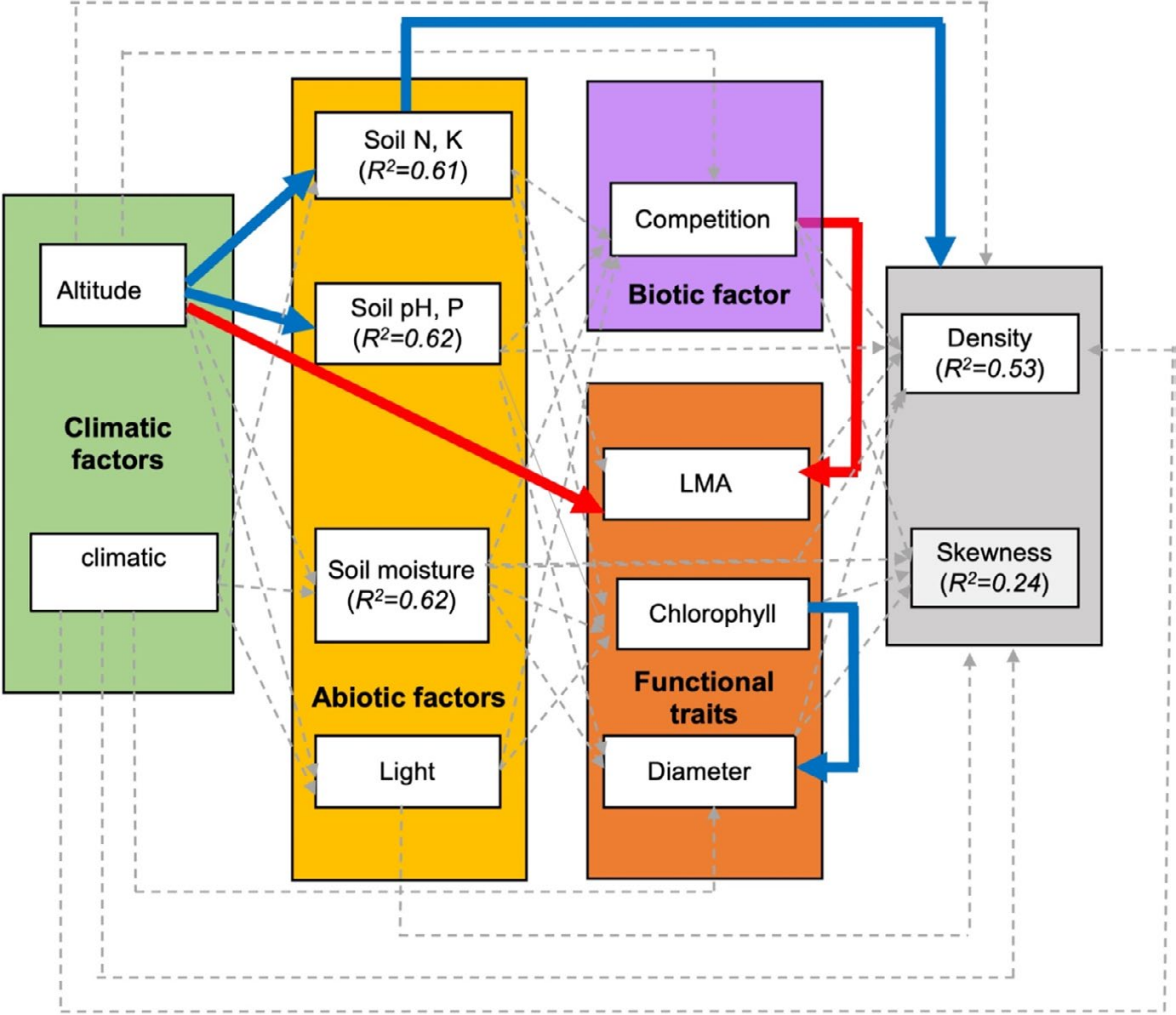
Structural equation model showing the distance from the centroid of climatic niche and altitude, affects *Thunbergia atacorensis* population density and population structure. Dashed arrow shows nonsignificant correlation, blue arrow means positive correlation, and red arrow shows negative correlations. *R* ^2^ represent the conditional coefficients of determination for each linear mixed effect. Soil N, P represent the first component of the PCA of soil fertility, while Soil pH, P represent the second component

**FIGURE 5:**
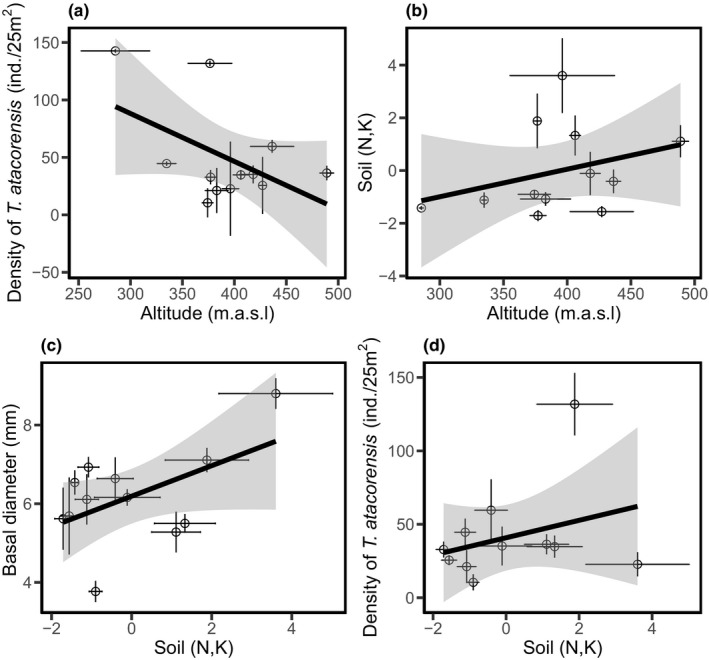
(a) Relationship between altitude and density per plot, (b) altitude and soil fertility (N, K), (c) Basal diameter and soil fertility (N, K), (d) Density per plot and soil fertility (N, K) . All these relationships were significant (*p* < .05)

We found no significant direct effects of geographic distance (*β* = 0.146 ± 0.176, *p* = .430, *R* ^2^ = .59, Figure 3) or climatic distance (*β* = −0.002 ± 0.001, *p* = .848, *R* ^2^ = .53, Figure 4) on *T*. *atacorensis* density. As expected, plant size was positively affected by leaf chlorophyll content (Figures 3,4). Similarly to plant density, the skewness of population structure was not significantly affected by climatic distance (*β* = −0.012 ± 0.008, *p* = .16, *R* ^2^ = .24, Figures 4, 5d) or geographical distance (*β* = −0.244 ± 0.134, *p* = .05, *R* ^2^ = .27, Figure 2). We found no direct effect of interspecific competition on population density (*β* = −0.031 ± 0.1139, *p* = .789, *R* ^2^ = .27, Figure 2). Soil pH and P increased with climatic distance (*β* = −0.054 ± 0.09, *p* = .033, *R* ^2^ = .62, Figure 2).

Surprisingly, we found no mediating effect of interspecific competition on population density, skewness, and basal diameter (Appendix S4, Tables S1, S2). Similarly, except for soil N and P, we found no mediating effect of geographic/climatic distance on other abiotic factors (soil moisture, pH, P, and light) or functional traits (LMA, chlorophyll content) (Figures 3, 4). In contrast, *T*. *atacorensis* density increased with soil N and K (*β* = 0.371 ± 0.082, *p* = .038, *R* ^2^ = .53, Figures 3, 5d) and soil N and K increased with altitude (*β* = 0.391 ± 0.302, *p* = .011, *R* ^2^ = .61, Figures 3, 5b). In addition, we found an indirect positive effect of altitude on plant size (basal diameter) mediated by the positive effect of soil N and K (Figures 3, 5b, c). Overall, the structural equation model that included climatic distance and altitude had better fit (AIC = 187.58) than the one that included geographic distance (AIC = 192.69). Including or removing the two isolated Sobakperou mountain populations from our models did not change our results (Appendix S2, Tables S1, S2).

## 4 DISCUSSION

### 4.1 No support for the center‐periphery hypothesis

Most tests of the center‐periphery hypothesis considered the geographical gradient as the main driver of the spatial variation of plant density. In this study, we used the geographic and climatic gradient and collected empirical data to advance our understanding of the ecological and evolutionary drivers of range limitation in endemic species. Using a tropical study system from a region where the center‐periphery hypothesis has rarely been tested, we found no support for the center‐periphery hypothesis using the geographic or climatic distance. These results add to increasing evidence that geographic or climatic distances by themselves do not influence species abundance. Particularly, Dallas et al. (2017) used a range of species including plants and animals to show that there was no significant variation in abundance across their geographic range and climatic niche. The authors suggested that species abundance could be driven by unmeasured factors such as biotic and abiotic factors, which may explain the failure to detect a significant decline in density from center to periphery. In our study, we measured several additional (a)biotic variables but still did not find support for the center‐periphery hypothesis. This lack of support for the center‐periphery hypothesis could also be due to mismatches between geographic distribution and the ecological niche as reported elsewhere (Pagel et al., 2020). Other possible causes include niche truncation caused by the lack of similar conditions in central and peripheral range due to geographic constraints (Papuga et al., 2018; Yañez et al., 2020), stabilizing selection at the periphery (Devictor et al., 2010) or dispersal limitation (Willi & Van Buskirk, 2019).

Previous studies showed that competition can synergistically interact with abiotic conditions to decrease population density at range's edge (Pulliam, 2000; Vergeer & Kunin, 2013). However, we found no mediating effect of most abiotic or biotic factors on the relationship between distance from climatic center and population density. Several mechanisms could explain such patterns. Environmental conditions at the center of climatic and geographic range may not be the most favorable to the species. Similarly, interspecific competition may be weaker at the center of the range than at periphery limiting its interactive influence. For example, we found that soil nitrogen and potassium increased with climatic distance but with no consequence on the spatial variation of interspecific competition. Beside competition, other potential biotic interactions such as herbivory or pollination which we did not measure in this study may be the drivers of the observed distribution of *T*. *atacorensis* populations.

### 4.2 Soil nutrients as an important driver of range limitation

Altitude had no direct influence on plant density and size. However, we found a strong direct positive effect of soil nutrients on plant density. Several studies showed that plant density decreases with altitude due to temperature variation and metabolic limitations at higher elevations (Angert, 2006; Souza et al., 2018). Instead of temperature gradient, for *T*. *atacorensis*, soil macronutrients variation along elevational gradient drives plant density. Particularly in tropical soils, phosphorus is a limiting factor (Camenzind et al., 2018; Hou et al., 2020). For instance, soil phosphorus decreased with altitude, and this can slow plant growth at higher elevations (Coomes & Allen, 2007). However, in our study, *T*. *atacorensis* individuals were larger at higher elevations due to high soil nitrogen and potassium concentrations but in low density. Such trade‐offs between large individual size and low density at higher elevations could be due to stronger intraspecific competition at higher elevations.

Overall, we found no support for the center‐periphery hypothesis that predicts that species density will decrease from center to the periphery of its range. Variation in soil properties along the elevation gradient drives the spatial distribution of our study species. Our finding that peripheral populations, with suboptimal climate, have similar population density suggests that for mountainous species in heterogenous landscapes, local ecological processes are stronger drivers of species distribution than macroscale climatic factors.

## CONFLICT OF INTEREST

The authors declare none.

## AUTHOR CONTRIBUTIONS

**Jacob K. Moutouama:** Conceptualization (lead); Data curation (lead); Formal analysis (lead); Funding acquisition (lead); Investigation (lead); Methodology (lead); Visualization (lead); Writing – original draft (lead); Writing – review & editing (lead). **Orou G. Gaoue:** Conceptualization (supporting); Data curation (supporting); Formal analysis (supporting); Funding acquisition (supporting); Methodology (supporting); Supervision (lead); Writing – original draft (supporting); Writing – review & editing (supporting).

### OPEN RESEARCH BADGES

This article has earned an Open Data and Open Materials Badges for making publicly available the digitally‐shareable data necessary to reproduce the reported results. The data is available at https://doi.org/10.5061/dryad.ghx3ffbqp and https://doi.org/10.5281/zenodo.5806806.

## Supporting information

Supplementary Material

## Data Availability

Data used for this manuscript are available and published online at Data Dryad https://doi.org/10.5061/dryad.ghx3ffbqp (Moutouama & Gaoue, 2022).
