# Supplementary material for: Altitude‐mediated soil properties, not geography or climatic distance, explain the distribution of a tropical endemic herb
Source: Ecol Evol. 2022 Feb 9;12(2):e8572. doi: 10.1002/ece3.8572 (PMC8826065; doi:10.1002/ece3.8572)
Supplement: Supplementary file 1 — Supplementary Material [file ECE3-12-e8572-s001.docx]

**Supplementary information**

**Appendix S1:**

Table S1. Occurrence records for populations sampled and the number of plots installed per population.

| Longitude | Latitude | Population | Number of plots installed |
| --- | --- | --- | --- |
| 1.34105 | 10.270279 | Poteye | 5 |
| 1.466705 | 10.294874 | Pouya | 5 |
| 1.499549 | 10.201108 | Kouaterna | 5 |
| 1.634746 | 10.68536 | Tandarfa | 5 |
| 1.269294 | 10.393611 | Marétingou | 5 |
| 1.209394 | 10.162479 | Koubirgou | 5 |
| 1.239261 | 10.146821 | kouyissikou | 5 |
| 1.192084 | 10.159625 | Kougnangou | 2 |
| 1.186998 | 10.1157 | Kouwetacoingou | 5 |
| 1.566943 | 9.977977 | Tassigourou | 5 |
| 2.160704 | 9.138537 | Sobakperou | 4 |
| 2.163432 | 9.127444 | Sesea | 3 |

**Appendix S2:**

**Figure S 1**: Conceptual framework. The diagram demonstrates the relationship between main predictions of the direct and indirect effects of distance from the center (geographic and climatic) and altitude on *Thunbergia atacorensis* population density, as well as population skewness mediated by abiotic and biotic factors. The line with one arrow illustrates a uni-directional relationship, while the solid line with double depicts a bi-directional relationship. Soil (N, K) represents mainly the effect of Nitrogen and Potassium in soil while Soil (N, K) represent the effect of Phosphorus and pH. Skewness represents the measure of asymmetry of basal diameter per population. Density is the abundance of *Thunbergia atacorensis* per 25 m^2^


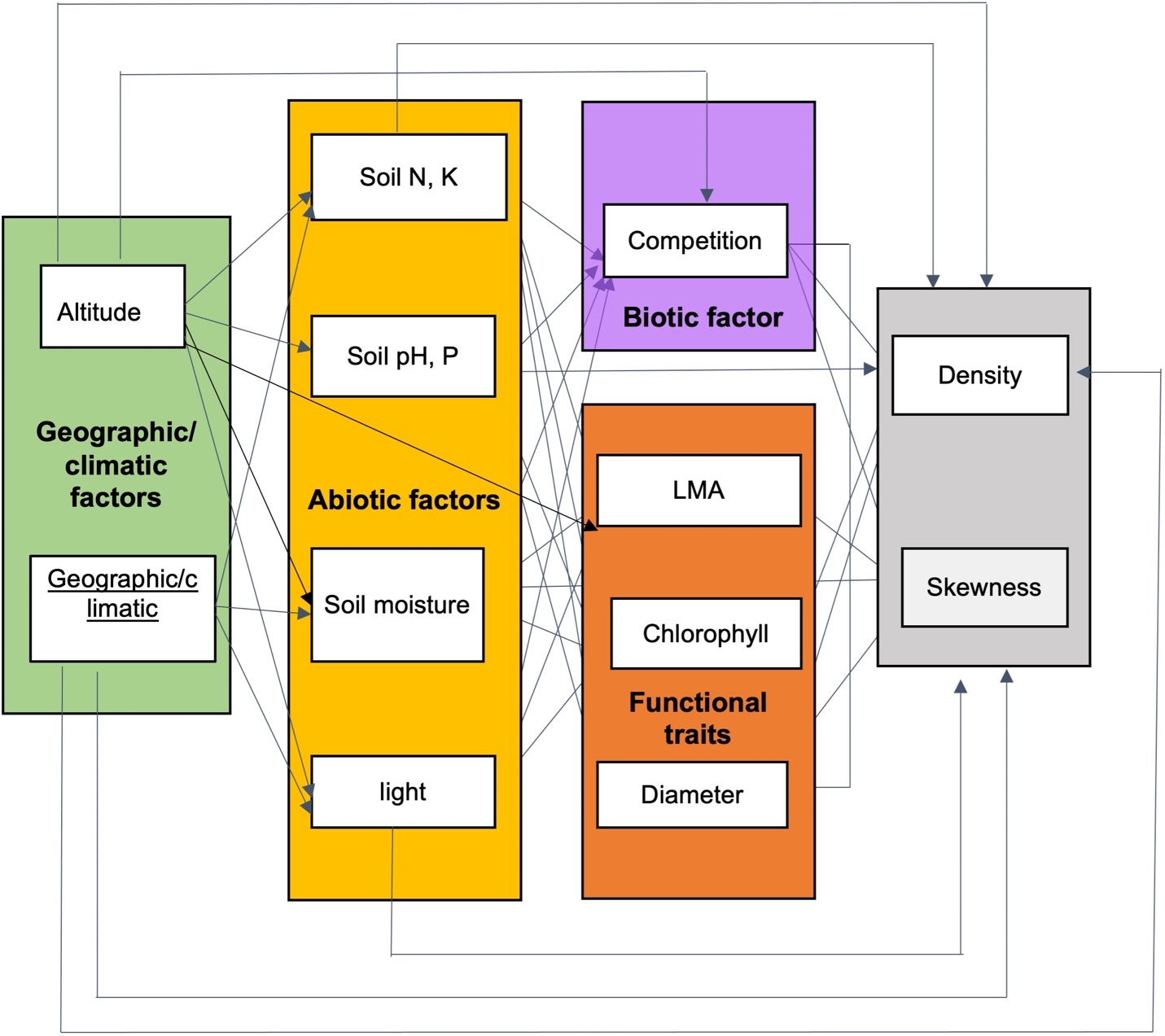


Table S1. The relationship between the distance from climatic center and altitude on population abundance and population skewness with the 10 Northern populations. PCA 1 is the first component of the principal component analysis and capture mainly the effect of Nitrogen and Potassium in soil while PCA 2 is the second component of the principal component analysis and represent the effect of Phosphorus and pH in soil. LMA is leaf mass per area. PAR is Photosynthetic active radiation. SRU is space resource utilization which is a proxy of inter specific competition. The “*” represent the level of significance of the relationship.

| Response | Predictor | | Estimate | SE | DF | Critical Value | P | Std Estimate |
| --- | --- | --- | --- | --- | --- | --- | --- | --- |
| PCA1 | | Geographic Distance | 0.1187 | 0.3372 | 35 | 0.3521 | 0.7269 | 0.0697 |
| PCA1 | | Altitude | 0.4194 | 0.3377 | 35 | 1.2421 | 0.2225 | 0.2415 |
| PCA2 | | Geographic Distance | 0.1102 | 0.3519 | 35 | 0.3132 | 0.756 | 0.0662 |
| PCA2 | | Altitude | 0.9487 | 0.3525 | 35 | 2.691 | 0.0108 | 0.5582 * |
| LMA | | PCA1 | 0.1838 | 0.071 | 29 | 2.5882 | 0.0149 | 0.3139 * |
| LMA | | Geographic Distance | 0.0029 | 0.1847 | 29 | 0.0154 | 0.9878 | 0.0029 |
| LMA | | PCA2 | 0.109 | 0.0703 | 29 | 1.5509 | 0.1318 | 0.1822 |
| LMA | | SRU | -0.1246 | 0.0906 | 29 | -1.375 | 0.1797 | -0.1236 |
| LMA | | PAR | 0.0471 | 0.1167 | 29 | 0.4036 | 0.6895 | 0.0468 |
| LMA | | Altitude | -0.4425 | 0.2099 | 29 | -2.1086 | 0.0437 | -0.4351 * |
| LMA | | Soil moisture | -0.0271 | 0.1237 | 29 | -0.2193 | 0.828 | -0.0272 |
| Chlorophyll | | PCA1 | 0.0466 | 0.0984 | 29 | 0.4736 | 0.6393 | 0.08 |
| Chlorophyll | | Geographic Distance | -0.0832 | 0.1971 | 29 | -0.422 | 0.6762 | -0.0838 |
| Chlorophyll | | PCA2 | -0.0341 | 0.0949 | 29 | -0.3587 | 0.7224 | -0.0572 |
| Chlorophyll | | SRU | 0.032 | 0.1356 | 29 | 0.2361 | 0.815 | 0.0319 |
| Chlorophyll | | PAR | 0.2365 | 0.1572 | 29 | 1.5044 | 0.1433 | 0.2362 |
| Chlorophyll | | Altitude | -0.436 | 0.212 | 29 | -2.0568 | 0.0488 | -0.4308 * |
| Chlorophyll | | Soil moisture | 0.0164 | 0.1696 | 29 | 0.0969 | 0.9234 | 0.0166 |
| PAR | | Geographic Distance | 0.0632 | 0.2032 | 34 | 0.3109 | 0.7578 | 0.0638 |
| PAR | | Altitude | 0 | 0.2098 | 34 | 0.0001 | 0.9999 | 0 |
| Soil moisture | | Geographic Distance | 0.3317 | 0.199 | 34 | 1.6664 | 0.1048 | 0.3311 |
| Soil moisture | | Altitude | 0.14 | 0.199 | 34 | 0.7035 | 0.4865 | 0.137 |
| Soil moisture | | SRU | 0.044 | 0.1294 | 34 | 0.34 | 0.7359 | 0.0435 |
| SRU | | PAR | 0.0999 | 0.1779 | 28 | 0.5615 | 0.5789 | 0.1001 |
| SRU | | Geographic Distance | 0.0554 | 0.1705 | 28 | 0.3249 | 0.7477 | 0.056 |
| SRU | | Altitude | 0.067 | 0.2086 | 28 | 0.3214 | 0.7503 | 0.0664 |
| SRU | | PCA1 | 0.0062 | 0.1051 | 28 | 0.0586 | 0.9537 | 0.0106 |
| SRU | | PCA2 | 0.0753 | 0.1029 | 28 | 0.7322 | 0.4701 | 0.1268 |
| SRU | | Chlorophyll | 0.1057 | 0.1822 | 28 | 0.5801 | 0.5665 | 0.106 |
| SRU | | LMA | -0.1341 | 0.1747 | 28 | -0.7673 | 0.4493 | -0.1351 |
| SRU | | Soil moisture | 0.1243 | 0.1906 | 28 | 0.6521 | 0.5196 | 0.1258 |
| diameter | | PAR | 0.0676 | 0.1314 | 26 | 0.514 | 0.6116 | 0.0713 |
| diameter | | Geographic Distance | 0.3218 | 0.1277 | 26 | 2.5189 | 0.0183 | 0.3430 * |
| diameter | | Altitude | 0.0845 | 0.1584 | 26 | 0.5336 | 0.5982 | 0.0883 |
| diameter | | PCA1 | 0.126 | 0.08 | 26 | 1.5751 | 0.1273 | 0.2287 |
| diameter | | PCA2 | 0.0532 | 0.0756 | 26 | 0.7041 | 0.4876 | 0.0944 |
| diameter | | SRU | -0.0878 | 0.1199 | 26 | -0.7324 | 0.4705 | -0.0926 |
| diameter | | Chlorophyll | 0.5735 | 0.1467 | 26 | 3.9089 | 0.0006 | 0.6065 *** |
| diameter | | LMA | -0.0528 | 0.1328 | 26 | -0.3976 | 0.6942 | -0.0561 |
| diameter | | density | 0.0495 | 0.1687 | 26 | 0.2932 | 0.7717 | 0.0465 |
| diameter | | Soil moisture | -0.1271 | 0.1475 | 26 | -0.8617 | 0.3967 | -0.1357 |
| density | | PAR | -0.0813 | 0.1379 | 25 | -0.5896 | 0.5607 | -0.0915 |
| density | | Geographic Distance | -0.0461 | 0.1722 | 25 | -0.2679 | 0.791 | -0.0523 |
| density | | Altitude | -0.2325 | 0.2013 | 25 | -1.1546 | 0.2592 | -0.2586 |
| density | | PCA1 | 0.1002 | 0.088 | 25 | 1.1381 | 0.2659 | 0.1936 |
| density | | PCA2 | -0.017 | 0.0802 | 25 | -0.2118 | 0.834 | -0.0321 |
| density | | SRU | -0.0308 | 0.1139 | 25 | -0.2704 | 0.7891 | -0.0346 |
| density | | Chlorophyll | 0.2408 | 0.1626 | 25 | 1.4814 | 0.151 | 0.2712 |
| density | | LMA | -0.2103 | 0.1612 | 25 | -1.3045 | 0.2039 | -0.2379 |
| density | | diameter | 0.008 | 0.1634 | 25 | 0.0489 | 0.9614 | 0.0085 |
| density | | Soil moisture | 0.3156 | 0.1451 | 25 | 2.1748 | 0.0393 | 0.3588 * |
| density | | skewness | 0.0792 | 0.1259 | 25 | 0.6289 | 0.5351 | 0.0888 |
| skewness | | PAR | 0.163 | 0.1618 | 26 | 1.0076 | 0.3229 | 0.1634 |
| skewness | | Geographic Distance | -0.242 | 0.1674 | 26 | -1.4461 | 0.1601 | -0.2449 |
| skewness | | Altitude | -0.0146 | 0.1891 | 26 | -0.0773 | 0.939 | -0.0145 |
| skewness | | PCA1 | -0.0044 | 0.0988 | 26 | -0.0447 | 0.9647 | -0.0076 |
| skewness | | PCA2 | -0.0249 | 0.0942 | 26 | -0.2648 | 0.7932 | -0.042 |
| skewness | | SRU | 0.0703 | 0.1495 | 26 | 0.4706 | 0.6419 | 0.0704 |
| skewness | | Chlorophyll | -0.226 | 0.2063 | 26 | -1.0955 | 0.2833 | -0.2268 |
| skewness | | LMA | 0.0713 | 0.1594 | 26 | 0.4472 | 0.6584 | 0.0719 |
| skewness | | diameter | -0.2655 | 0.209 | 26 | -1.2704 | 0.2152 | -0.252 |
| skewness | | Soil moisture | -0.2569 | 0.1745 | 26 | -1.4717 | 0.1531 | -0.2604 |

Table S2. The relationship between the distance from climatic center and altitude on population abundance and population skewness with the 10 Northern populations. PCA 1 is the first component of the principal component analysis and capture mainly the effect of Nitrogen and Potassium in soil while PCA 2 is the second component of the principal component analysis and represent the effect of Phosphorus and pH in soil. LMA is leaf mass per area. PAR is Photosynthetic active radiation. SRU is space resource utilization which is a proxy of inter specific competition. The “*” represent the level of significance of the relationship.

| Response | Predictor | Estimate | SE | DF | Critical Value | P | Std Estimate |
| --- | --- | --- | --- | --- | --- | --- | --- |
| PCA1 | Climatic Distance | -0.0107 | 0.0184 | 35 | -0.5832 | 0.5635 | -0.0063 |
| PCA1 | Altitude | 0.3786 | 0.3072 | 35 | 1.2327 | 0.2259 | 0.218 |
| PCA2 | Climatic Distance | 0.0545 | 0.019 | 35 | 2.8597 | 0.0071 | 0.0327 ** |
| PCA2 | Altitude | 0.9916 | 0.3082 | 35 | 3.2174 | 0.0028 | 0.5835 ** |
| LMA | PCA1 | 0.1927 | 0.0724 | 29 | 2.6604 | 0.0126 | 0.3291 * |
| LMA | Climatic Distance | 0.0058 | 0.0111 | 29 | 0.5254 | 0.6033 | 0.0059 |
| LMA | PCA2 | 0.0912 | 0.0775 | 29 | 1.1775 | 0.2486 | 0.1524 |
| LMA | SRU | -0.1279 | 0.0903 | 29 | -1.416 | 0.1674 | -0.1269 |
| LMA | PAR | 0.0546 | 0.1166 | 29 | 0.4679 | 0.6433 | 0.0542 |
| LMA | Altitude | -0.4372 | 0.1843 | 29 | -2.3723 | 0.0245 | -0.4298 * |
| LMA | Soil moisture | -0.0404 | 0.1216 | 29 | -0.3319 | 0.7424 | -0.0405 |
| Chlorophyll | PCA1 | 0.0378 | 0.0993 | 29 | 0.3803 | 0.7065 | 0.0648 |
| Chlorophyll | Climatic Distance | -0.0073 | 0.0111 | 29 | -0.656 | 0.517 | -0.0074 |
| Chlorophyll | PCA2 | -0.0138 | 0.1009 | 29 | -0.1373 | 0.8918 | -0.0233 |
| Chlorophyll | SRU | 0.0355 | 0.1354 | 29 | 0.262 | 0.7952 | 0.0354 |
| Chlorophyll | PAR | 0.2345 | 0.1567 | 29 | 1.4962 | 0.1454 | 0.2342 |
| Chlorophyll | Altitude | -0.4158 | 0.1973 | 29 | -2.1075 | 0.0438 | -0.4108 * |
| Chlorophyll | Soil moisture | 0.0154 | 0.166 | 29 | 0.093 | 0.9265 | 0.0156 |
| PAR | Climatic Distance | -0.0033 | 0.0113 | 34 | -0.2888 | 0.7745 | -0.0033 |
| PAR | Altitude | -0.0359 | 0.1973 | 34 | -0.1818 | 0.8568 | -0.0355 |
| Soil moisture | Climatic Distance | 0.003 | 0.0108 | 35 | 0.2787 | 0.7821 | 0.003 |
| Soil moisture | Altitude | 0.0371 | 0.1854 | 35 | 0.2003 | 0.8424 | 0.0363 |
| SRU | PAR | 0.0986 | 0.1776 | 28 | 0.5554 | 0.5831 | 0.0988 |
| SRU | Climatic Distance | 0.004 | 0.0093 | 28 | 0.4351 | 0.6668 | 0.0041 |
| SRU | Altitude | 0.0593 | 0.2004 | 28 | 0.2959 | 0.7695 | 0.0587 |
| SRU | PCA1 | 0.0085 | 0.1052 | 28 | 0.0808 | 0.9362 | 0.0146 |
| SRU | PCA2 | 0.0654 | 0.1068 | 28 | 0.6122 | 0.5453 | 0.1101 |
| SRU | Chlorophyll | 0.1114 | 0.183 | 28 | 0.609 | 0.5474 | 0.1117 |
| SRU | LMA | -0.1391 | 0.1752 | 28 | -0.7943 | 0.4337 | -0.1402 |
| SRU | Soil moisture | 0.1265 | 0.1873 | 28 | 0.6755 | 0.5049 | 0.1281 |
| diameter | PAR | 0.0743 | 0.1305 | 26 | 0.5694 | 0.574 | 0.0785 |
| diameter | Climatic Distance | 0.0185 | 0.0071 | 26 | 2.6211 | 0.0144 | 0.0198 * |
| diameter | Altitude | 0.027 | 0.1514 | 26 | 0.1784 | 0.8598 | 0.0282 |
| diameter | PCA1 | 0.1305 | 0.0797 | 26 | 1.6373 | 0.1136 | 0.2368 |
| diameter | PCA2 | 0.0114 | 0.0782 | 26 | 0.1458 | 0.8852 | 0.0202 |
| diameter | SRU | -0.0956 | 0.1189 | 26 | -0.8039 | 0.4287 | -0.1008 |
| diameter | Chlorophyll | 0.5787 | 0.1454 | 26 | 3.9792 | 0.0005 | 0.6119 *** |
| diameter | LMA | -0.0698 | 0.1328 | 26 | -0.5257 | 0.6036 | -0.0742 |
| diameter | density | 0.0685 | 0.169 | 26 | 0.4054 | 0.6885 | 0.0644 |
| diameter | Soil moisture | -0.1043 | 0.1438 | 26 | -0.7252 | 0.4748 | -0.1113 |
| density | PAR | -0.0713 | 0.1331 | 26 | -0.5357 | 0.5967 | -0.0801 |
| density | Climatic Distance | -0.0089 | 0.0099 | 26 | -0.8964 | 0.3783 | -0.0101 |
| density | Altitude | -0.2254 | 0.1875 | 26 | -1.2023 | 0.2401 | -0.2507 |
| density | PCA1 | 0.0779 | 0.0888 | 26 | 0.8772 | 0.3884 | 0.1505 |
| density | PCA2 | 0.0052 | 0.0837 | 26 | 0.0625 | 0.9507 | 0.0099 |
| density | SRU | -0.0178 | 0.1116 | 26 | -0.1593 | 0.8747 | -0.02 |
| density | Chlorophyll | 0.1903 | 0.1574 | 26 | 1.2089 | 0.2376 | 0.2143 |
| density | LMA | -0.1876 | 0.1592 | 26 | -1.1778 | 0.2496 | -0.2122 |
| density | diameter | 0.0117 | 0.1584 | 26 | 0.0737 | 0.9418 | 0.0124 |
| density | Soil moisture | 0.3064 | 0.1352 | 26 | 2.2665 | 0.032 | 0.3483 * |
| skewness | PAR | 0.1565 | 0.1642 | 26 | 0.9528 | 0.3495 | 0.1569 |
| skewness | Climatic Distance | -0.0091 | 0.0094 | 26 | -0.9683 | 0.3418 | -0.0092 |
| skewness | Altitude | 0.046 | 0.1841 | 26 | 0.2501 | 0.8045 | 0.0457 |
| skewness | PCA1 | 0.003 | 0.101 | 26 | 0.0292 | 0.9769 | 0.0051 |
| skewness | PCA2 | -0.0081 | 0.0986 | 26 | -0.0823 | 0.9351 | -0.0137 |
| skewness | SRU | 0.0669 | 0.1522 | 26 | 0.4393 | 0.6641 | 0.0669 |
| skewness | Chlorophyll | -0.1986 | 0.2126 | 26 | -0.9345 | 0.3586 | -0.1994 |
| skewness | LMA | 0.0764 | 0.1631 | 26 | 0.4683 | 0.6435 | 0.077 |
| skewness | diameter | -0.2993 | 0.2139 | 26 | -1.3993 | 0.1735 | -0.2841 |
| skewness | Soil moisture | -0.2931 | 0.1739 | 26 | -1.6852 | 0.1039 | -0.297 |

**Appendix S3:**

Figure S 1: Principal component analysis of soil fertility data. Here the two first components explain more than 54% of variability of soil fertility. Nitrogen and potassuim are more associated with the first component and the pH and Phosphore are more associated to the second component.


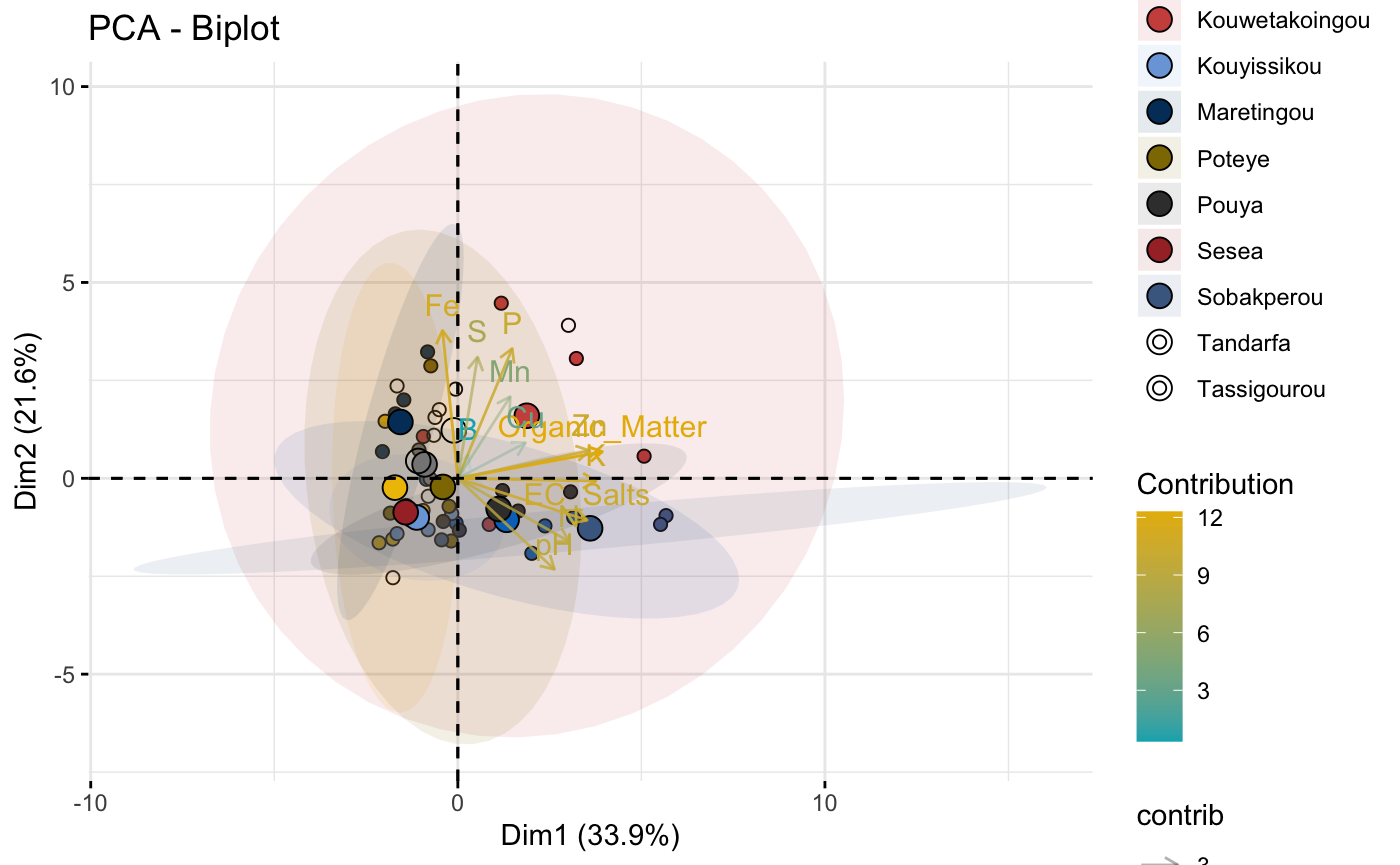


**Appendix S4:**

Table S1. The relationship between the distance from the geographical center and altitude on population abundance and population skewness. The “*” represent the level of significance of the relationship.

| Response | Predictor | Estimate | SE | DF | Critical Value | P | Std Estimate |
| --- | --- | --- | --- | --- | --- | --- | --- |
| PCA1 | Geographic Distance | 0.8557 | 0.3084 | 40 | 2.7748 | 0.0084 | 0.4057** |
| PCA1 | Altitude | 0.883 | 0.2822 | 40 | 3.1292 | 0.0033 | 0.4069** |
| PCA2 | Geographic Distance | 0.3781 | 0.2831 | 40 | 1.3356 | 0.1892 | 0.2343 |
| PCA2 | Altitude | 0.73 | 0.266 | 40 | 2.7441 | 0.009 | 0.4397** |
| LMA | PCA1 | 0.1011 | 0.0748 | 30 | 1.3515 | 0.1866 | 0.2139 |
| LMA | Geographic Distance | 0.1744 | 0.1661 | 30 | 1.0502 | 0.302 | 0.1749 |
| LMA | PCA2 | -0.0078 | 0.075 | 30 | -0.1035 | 0.9183 | -0.0126 |
| LMA | SRU | -0.1085 | 0.0977 | 30 | -1.1102 | 0.2757 | -0.1108 |
| LMA | PAR | 0.2849 | 0.1114 | 30 | 2.5581 | 0.0158 | 0.2908* |
| LMA | Altitude | -0.2769 | 0.1819 | 30 | -1.5223 | 0.1384 | -0.2698 |
| LMA | Soil moisture | -0.1692 | 0.138 | 30 | -1.2259 | 0.2298 | -0.1618 |
| Chlorophyll | PCA1 | 0.0759 | 0.0848 | 34 | 0.8949 | 0.3771 | 0.1651 |
| Chlorophyll | Geographic Distance | -0.3903 | 0.1751 | 34 | -2.2292 | 0.0325 | -0.4023* |
| Chlorophyll | PCA2 | -0.004 | 0.0882 | 34 | -0.0448 | 0.9645 | -0.0066 |
| Chlorophyll | SRU | 0.1418 | 0.1253 | 34 | 1.1316 | 0.2657 | 0.1489 |
| Chlorophyll | PAR | 0.1848 | 0.1348 | 34 | 1.371 | 0.1794 | 0.194 |
| Chlorophyll | Altitude | -0.5078 | 0.1909 | 34 | -2.6605 | 0.0118 | -0.5088* |
| Chlorophyll | Soil moisture | -0.0958 | 0.1534 | 34 | -0.6245 | 0.5365 | -0.0942 |
| PAR | Geographic Distance | -0.0705 | 0.1753 | 39 | -0.4023 | 0.6896 | -0.0693 |
| PAR | Altitude | 0.0861 | 0.1726 | 39 | 0.4987 | 0.6208 | 0.0821 |
| Soil moisture | Geographic Distance | -0.0845 | 0.1811 | 39 | -0.4667 | 0.6433 | -0.0886 |
| Soil moisture | Altitude | -0.2879 | 0.1639 | 39 | -1.7567 | 0.0868 | -0.2933 |
| Soil moisture | SRU | -0.0009 | 0.1142 | 39 | -0.0079 | 0.9938 | -0.001 |
| SRU | PAR | -0.0392 | 0.1761 | 29 | -0.2229 | 0.8252 | -0.0392 |
| SRU | Geographic Distance | 0.1802 | 0.1927 | 29 | 0.935 | 0.3575 | 0.1769 |
| SRU | Altitude | -0.1125 | 0.2322 | 29 | -0.4843 | 0.6318 | -0.1073 |
| SRU | PCA1 | -0.0814 | 0.1008 | 29 | -0.807 | 0.4263 | -0.1685 |
| SRU | PCA2 | 0.0917 | 0.1073 | 29 | 0.8542 | 0.4 | 0.1452 |
| SRU | Chlorophyll | 0.1802 | 0.1881 | 29 | 0.958 | 0.346 | 0.1716 |
| SRU | LMA | -0.1823 | 0.1909 | 29 | -0.9548 | 0.3476 | -0.1785 |
| SRU | Soil moisture | 0.2998 | 0.2188 | 29 | 1.37 | 0.1812 | 0.2807 |
| diameter | PAR | 0.1135 | 0.1187 | 27 | 0.956 | 0.3475 | 0.1332 |
| diameter | Geographic Distance | 0.4316 | 0.1319 | 27 | 3.2716 | 0.0029 | 0.4976** |
| diameter | Altitude | -0.0537 | 0.1714 | 27 | -0.3133 | 0.7565 | -0.0602 |
| diameter | PCA1 | 0.1256 | 0.0692 | 27 | 1.816 | 0.0805 | 0.3055 |
| diameter | PCA2 | 0.0053 | 0.0725 | 27 | 0.0729 | 0.9424 | 0.0098 |
| diameter | SRU | -0.1438 | 0.1066 | 27 | -1.3495 | 0.1884 | -0.1689 |
| diameter | Chlorophyll | 0.4356 | 0.129 | 27 | 3.3778 | 0.0022 | 0.4871** |
| diameter | LMA | -0.0977 | 0.1309 | 27 | -0.7463 | 0.4619 | -0.1123 |
| diameter | density | -0.1678 | 0.1512 | 27 | -1.1094 | 0.277 | -0.1684 |
| diameter | Soil moisture | 0.0973 | 0.1565 | 27 | 0.6217 | 0.5393 | 0.107 |
| density | PAR | -0.0367 | 0.1279 | 26 | -0.2868 | 0.7765 | -0.0429 |
| density | Geographic Distance | -0.085 | 0.1782 | 26 | -0.4769 | 0.6374 | -0.0976 |
| density | Altitude | -0.3908 | 0.1903 | 26 | -2.0542 | 0.0501 | -0.4363 |
| density | PCA1 | 0.0485 | 0.0791 | 26 | 0.6138 | 0.5446 | 0.1176 |
| density | PCA2 | 0.001 | 0.0767 | 26 | 0.013 | 0.9897 | 0.0018 |
| density | SRU | 0.0057 | 0.1047 | 26 | 0.0542 | 0.9572 | 0.0066 |
| density | Chlorophyll | 0.0962 | 0.1485 | 26 | 0.6475 | 0.523 | 0.1071 |
| density | LMA | -0.0956 | 0.16 | 26 | -0.5978 | 0.5551 | -0.1096 |
| density | diameter | -0.1047 | 0.1597 | 26 | -0.6559 | 0.5177 | -0.1043 |
| density | Soil moisture | 0.3447 | 0.1468 | 26 | 2.3474 | 0.0268 | 0.3776* |
| density | skewness | 0.0024 | 0.1221 | 26 | 0.0199 | 0.9843 | 0.0027 |
| skewness | PAR | 0.1795 | 0.1583 | 27 | 1.1338 | 0.2669 | 0.1864 |
| skewness | Geographic Distance | -0.0649 | 0.1999 | 27 | -0.3246 | 0.748 | -0.0662 |
| skewness | Altitude | -0.0206 | 0.2108 | 27 | -0.0979 | 0.9227 | -0.0205 |
| skewness | PCA1 | -0.0316 | 0.0942 | 27 | -0.3353 | 0.74 | -0.068 |
| skewness | PCA2 | 0.043 | 0.0953 | 27 | 0.4507 | 0.6558 | 0.0707 |
| skewness | SRU | -0.0098 | 0.1416 | 27 | -0.0694 | 0.9452 | -0.0102 |
| skewness | Chlorophyll | -0.335 | 0.186 | 27 | -1.801 | 0.0829 | -0.3313 |
| skewness | LMA | 0.2251 | 0.1765 | 27 | 1.275 | 0.2132 | 0.2289 |
| skewness | diameter | -0.3771 | 0.2105 | 27 | -1.7914 | 0.0844 | -0.3336 |
| skewness | Soil moisture | -0.1737 | 0.1938 | 27 | -0.8963 | 0.378 | -0.1689 |

Table S2. The relationship between the distance from the climatic center and altitude on population abundance and population skewness. The “*” represent the level of significance of the relationship.

| Response | Predictor | Estimate | SE | DF | Critical Value | P | Std Estimate |
| --- | --- | --- | --- | --- | --- | --- | --- |
| PCA1 | Climatic Distance | 0.0121 | 0.0149 | 40 | 0.8149 | 0.4199 | 0.4014** |
| PCA1 | Altitude | 0.8711 | 0.3163 | 40 | 2.7539 | 0.0088 | 0.4014** |
| PCA2 | Climatic Distance | -0.0006 | 0.0126 | 40 | -0.0483 | 0.9617 | -0.0089 |
| PCA2 | Altitude | 0.626 | 0.2698 | 40 | 2.32 | 0.0255 | 0.3771* |
| LMA | PCA1 | 0.1327 | 0.0696 | 30 | 1.907 | 0.0661 | 0.2806 |
| LMA | Climatic Distance | 0.0008 | 0.007 | 30 | 0.108 | 0.9147 | 0.0179 |
| LMA | PCA2 | 0.0057 | 0.075 | 30 | 0.0755 | 0.9403 | 0.0092 |
| LMA | SRU | -0.1198 | 0.0987 | 30 | -1.2147 | 0.234 | -0.1224 |
| LMA | PAR | 0.2801 | 0.1129 | 30 | 2.4805 | 0.019 | 0.286* |
| LMA | Altitude | -0.3191 | 0.1897 | 30 | -1.6826 | 0.1028 | -0.311 |
| LMA | Soil moisture | -0.1959 | 0.1425 | 30 | -1.3748 | 0.1794 | -0.1874 |
| Chlorophyll | PCA1 | 0.0063 | 0.0835 | 34 | 0.0755 | 0.9403 | 0.0137 |
| Chlorophyll | Climatic Distance | 0.0003 | 0.0078 | 34 | 0.0426 | 0.9662 | 0.0081 |
| Chlorophyll | PCA2 | -0.0388 | 0.0921 | 34 | -0.4209 | 0.6765 | -0.0645 |
| Chlorophyll | SRU | 0.1441 | 0.1309 | 34 | 1.1009 | 0.2787 | 0.1514 |
| Chlorophyll | PAR | 0.169 | 0.1426 | 34 | 1.1849 | 0.2443 | 0.1774 |
| Chlorophyll | Altitude | -0.3299 | 0.1937 | 34 | -1.7027 | 0.0978 | -0.3305 |
| Chlorophyll | Soil moisture | -0.0588 | 0.1641 | 34 | -0.3583 | 0.7223 | -0.0578 |
| PAR | Climatic Distance | 0.0038 | 0.0077 | 39 | 0.4934 | 0.6245 | 0.0877 |
| PAR | Altitude | 0.1255 | 0.1688 | 39 | 0.7438 | 0.4615 | 0.1198 |
| Soil moisture | Climatic Distance | 0.011 | 0.0079 | 40 | 1.4003 | 0.1691 | 0.2737 |
| Soil moisture | Altitude | -0.2125 | 0.1673 | 40 | -1.27 | 0.2114 | -0.2166 |
| SRU | PAR | -0.0355 | 0.182 | 29 | -0.1951 | 0.8467 | -0.0355 |
| SRU | Climatic Distance | -0.0044 | 0.0078 | 29 | -0.5609 | 0.5792 | -0.1019 |
| SRU | Altitude | -0.2453 | 0.2269 | 29 | -1.0812 | 0.2885 | -0.234 |
| SRU | PCA1 | -0.0468 | 0.1012 | 29 | -0.4621 | 0.6475 | -0.0969 |
| SRU | PCA2 | 0.1203 | 0.1101 | 29 | 1.0929 | 0.2834 | 0.1905 |
| SRU | Chlorophyll | 0.1095 | 0.1796 | 29 | 0.61 | 0.5466 | 0.1043 |
| SRU | LMA | -0.1797 | 0.2029 | 29 | -0.8857 | 0.3831 | -0.1759 |
| SRU | Soil moisture | 0.2791 | 0.227 | 29 | 1.2292 | 0.2289 | 0.2613 |
| diameter | PAR | 0.1333 | 0.1367 | 27 | 0.9752 | 0.3381 | 0.1565 |
| diameter | Climatic Distance | 0.0041 | 0.0065 | 27 | 0.6244 | 0.5376 | 0.1105 |
| diameter | Altitude | -0.2019 | 0.2 | 27 | -1.0095 | 0.3217 | -0.2262 |
| diameter | PCA1 | 0.2019 | 0.0768 | 27 | 2.6279 | 0.014 | 0.4909* |
| diameter | PCA2 | -0.0037 | 0.083 | 27 | -0.0441 | 0.9652 | -0.0068 |
| diameter | SRU | -0.0979 | 0.1169 | 27 | -0.8376 | 0.4096 | -0.115 |
| diameter | Chlorophyll | 0.3258 | 0.1336 | 27 | 2.438 | 0.0216 | 0.3643* |
| diameter | LMA | -0.0412 | 0.1605 | 27 | -0.2565 | 0.7995 | -0.0473 |
| diameter | density | -0.1662 | 0.1823 | 27 | -0.9117 | 0.37 | -0.1668 |
| diameter | Soil moisture | 0.0255 | 0.1827 | 27 | 0.1394 | 0.8902 | 0.028 |
| density | PAR | -0.0383 | 0.1235 | 27 | -0.31 | 0.759 | -0.0448 |
| density | Climatic Distance | -0.006 | 0.0061 | 27 | -0.9845 | 0.3336 | -0.1618 |
| density | Altitude | -0.437 | 0.1733 | 27 | -2.521 | 0.0179 | -0.4878* |
| density | PCA1 | 0.0409 | 0.0748 | 27 | 0.5464 | 0.5893 | 0.099 |
| density | PCA2 | 0.0111 | 0.0741 | 27 | 0.15 | 0.8819 | 0.0206 |
| density | SRU | -0.0021 | 0.1042 | 27 | -0.0201 | 0.9841 | -0.0025 |
| density | Chlorophyll | 0.1369 | 0.1256 | 27 | 1.0901 | 0.2853 | 0.1525 |
| density | LMA | -0.106 | 0.1477 | 27 | -0.7182 | 0.4788 | -0.1215 |
| density | diameter | -0.1309 | 0.144 | 27 | -0.9088 | 0.3715 | -0.1304 |
| density | Soil moisture | 0.3759 | 0.1482 | 27 | 2.5358 | 0.0173 | 0.4118* |
| skewness | PAR | 0.1633 | 0.144 | 27 | 1.1337 | 0.2669 | 0.1695 |
| skewness | Climatic Distance | -0.0128 | 0.0058 | 27 | -2.2067 | 0.036 | -0.308* |
| skewness | Altitude | -0.0528 | 0.1722 | 27 | -0.3069 | 0.7612 | -0.0524 |
| skewness | PCA1 | -0.0733 | 0.0869 | 27 | -0.844 | 0.4061 | -0.1577 |
| skewness | PCA2 | 0.064 | 0.0884 | 27 | 0.7239 | 0.4753 | 0.1053 |
| skewness | SRU | -0.0283 | 0.1305 | 27 | -0.2172 | 0.8297 | -0.0294 |
| skewness | Chlorophyll | -0.3419 | 0.1537 | 27 | -2.2245 | 0.0347 | -0.3382* |
| skewness | LMA | 0.2105 | 0.1514 | 27 | 1.3899 | 0.1759 | 0.2141 |
| skewness | diameter | -0.3548 | 0.177 | 27 | -2.0044 | 0.0551 | -0.3139 |
| skewness | Soil moisture | -0.0658 | 0.1885 | 27 | -0.3488 | 0.7299 | -0.064 |
